# Effect of practical training on the learning motivation profile of Japanese pharmacy students using structural equation modeling

**DOI:** 10.3352/jeehp.2017.14.2

**Published:** 2017-02-07

**Authors:** Shigeo Yamamura, Rieko Takehira

**Affiliations:** 1 Faculty of Pharmaceutical Sciences, Josai International University, Chiba, Japan; 2 Kitasato University School of pharmacy, Tokyo, Japan; Hallym University, Korea

**Keywords:** Pharmacy students, Learning, Motivation, Training

## Abstract

**Purpose:**

To establish a model of Japanese pharmacy students’ learning motivation profile and investigate the effects of pharmaceutical practical training programs on their learning motivation.

**Methods:**

The Science Motivation Questionnaire II was administered to pharmacy students in their 4th (before practical training), 5th (before practical training at clinical sites), and 6th (after all practical training) years of study at Josai International University in April, 2016. Factor analysis and multiple-group structural equation modeling were conducted for data analysis.

**Results:**

A total of 165 students participated. The learning motivation profile was modeled with 4 factors (intrinsic, career, self-determination, and grade motivation), and the most effective learning motivation was grade motivation. In the multiple-group analysis, the fit of the model with the data was acceptable, and the estimated mean value of the factor of ‘self-determination’ in the learning motivation profile increased after the practical training programs (P= 0.048, Cohen’s *d*= 0.43).

**Conclusion:**

Practical training programs in a 6-year course were effective for increasing learning motivation, based on ‘self-determination’ among Japanese pharmacy students. The results suggest that practical training programs are meaningful not only for providing clinical experience but also for raising learning motivation.

## Introduction

Japanese pharmacies and pharmacists are expected to make more contribution to healthcare in future super-aging society. In 2006, Japan decided to extend its 4-year pharmacist training course to a 6-year course to meet the social need for pharmacists and help improve the health of the community [1]. Along with extending the course term, some practical pharmacy training programs were introduced into the core curricula: pre-practical training including computer-based testing and objective structured clinical examination for 4th-year students, and practical training for 22 weeks in community pharmacies and hospital pharmacies for 5th-year students. Change in education program would have influence on the learning motivation of students. Regarding the relationship between motivation and education in health professions, systematic reviews based on self-determination theory have been published. They indicated that the students’ motivation was affected by educational environment [2,3]. As pharmacy students, they need to maintain high learning motivation to continue learning over a long period and become good professionals. Studies have focused on the learning motivation profile of medical students, but those on the profile of pharmacy students are sparse [4]. Yamaguchi et al. [4], reported the practical training increased the learning motivation of Japanese pharmacy students, but they did not mention the subscale of the motivation.

We hypothesized that a newly introduced practical training program in a 6-year course not just provides practical experience at clinical sites but can raise or change the learning motivation profile of Japanese pharmacy students. To verify our hypothesis, we used the Science Motivation Questionnaire II (SMQ-II) to establish the learning motivation profile of pharmacy students [5]. The SMQ-II was developed by Glynn et al. [5], and it assesses 5 components (intrinsic, career, self-determination, self-efficacy, and grade motivation) in students’ motivations to learn science. In this study, we used the SMQ-II questionnaire to establish a model for the learning motivation profile of Japanese pharmacy students and examine the effect of practical training programs on the learning motivation profile. Use of this measurement tool was permitted by Dr. Shawn M. Glynn, Josiah Meigs Distinguished Teaching Professor Emeritus, University of Georgia.

## Methods

### Study design

A cross-sectional study design was used.

### Materials and subjects

All pharmacy students except for those in a leave of absence from school in their 4th- to 6th-year in Josai International (171 students) were invited to participate. The survey was conducted in early April (the first semester starts in April in Japan) in 2016. The purpose of the survey was briefly explained to the students, and they were given instructions on how to complete the questionnaire. Students who were willing to participate in the survey signed a consent form and then answered the questions using an optical mark recognition sheet.

### Questionnaire

Students’ motivation profile was measured with the 25-item SMQ-II, which assesses the factors of students’ motivation (intrinsic, career, self-determination, self-efficacy, and grade motivation) to learn science [5]. The SMQ-II was also applied to evaluate the motivation to learn other subjects. A Japanese version of SMQ-II was used after replacing ‘science’ with ‘pharmacy’ and making corrections for minor character errors [6].

Responses were marked in Likert scale from 1 to 5: 1= never, 2= rarely, 3= sometimes, 4= usually, and 5= always.

### Statistics

Average scores and standard deviations were calculated for each item. Factor analysis was conducted to establish a basic model to express learning motivation using 25 items in the SMQ-II [5]. The maximum likelihood method was applied to find factors. Promax rotation as oblique rotation was employed since all factors were considered to be partially dependent on each other. The number of factors was determined by a scree plot and Kaiser-Guttmann criteria. To build a factor of the learning motivation, we extracted items for each subscale if they were loaded ≥ 0.4 on a particular factor but were < 0.4 on all other factors. Items loaded < 0.4 on all factors were removed from the item set, and factor analysis was repeated until all items were loaded ≥ 0.4 on one particular factor. The fit of the model with the data was examined in terms of chi-square, and Cronbach’s alpha was calculated to examine internal reliability.

Structural equation modeling (SEM) was used to establish a more reasonable model for the learning motivation profile of Japanese pharmacy students. SEM is a multivariate statistical method with or without latent variables like factor analysis and more flexible ways to establish complex models [7]. SEM is frequently used in social science and educational research [8] and is also applied to the study of motivation styles for learning [8,9].

In cases where heterogeneity in the learning motivation profile according to school year may be expected, a multiple-group SEM approach taking mean structure into account would be feasible [7]. A multiple-group analysis was applied to determine the changes in the mean values of latent variables (learning motivation factor) in the learning motivation profile between school years.

The fit of each model with the data was examined using several goodness-of-fit (GOF) statistics, such as chi-square, goodness-of-fit index (GFI), adjusted goodness-of-fit (AGFI), root mean square error of approximation (RMSEA), comparative fit index (CFI), and Akaike information criteria [7].

Factor analysis and SEM were carried out using IBM SPSS ver. 23.0 (IBM Co., Armonk, NY, USA) and AMOS ver. 23.0 (IBM Co.), respectively.

### Ethical approval

The survey was approved by the ethics committee of the Faculty of Pharmaceutical Sciences at Josai International University (the ID of the approval: 45).

## Results

In the survey, 165 completed responses were obtained and used in the analysis: 65 (female 39, male 26) out of 69 from the 4th-year students, 43 (female 24, male 19) out of 45 from the 5th-year students, and 57 (female 28, male 29) out of 57 from the 6th-year students. Overall, the effective response rate was 93.2%. Most of 4th-, 5th-, and 6th-year students were 22, 23, and 24 years old, respectively (Suppl. 1).

The average scores and standard deviations obtained for each item in SMQ-II [5] for each school year are summarized in Table 1. For several items, a significant difference (P< 0.05) was found in the average score, but no clear trend according to the school year was found.

Table 2 shows the results of the factor analysis. Statistical measures for sampling adequacy and internal consistency of each factor indicated whether the model was acceptable. This result indicates the model of learning motivation in Japanese pharmacy students was established using SMQ-II [5]. However, the established model consisted of 4 factors, not 5, in the SMQ-II [5]. The motivation based on ‘self-efficacy’ was not a factor of the learning motivation profile in Japanese pharmacy students. The most effective learning motivation factor was ‘grade motivation’ among Japanese pharmacy students. About 66.4% of variance could be explained by the 4 factors in the model obtained by factor analysis.

In the next step, we used SEM to explore a better model fit and express the learning motivation profile of Japanese pharmacy students. Taking the model with factor analysis as the basic model, we modified the model using a trial-and-error approach.

**Fig. 1.**
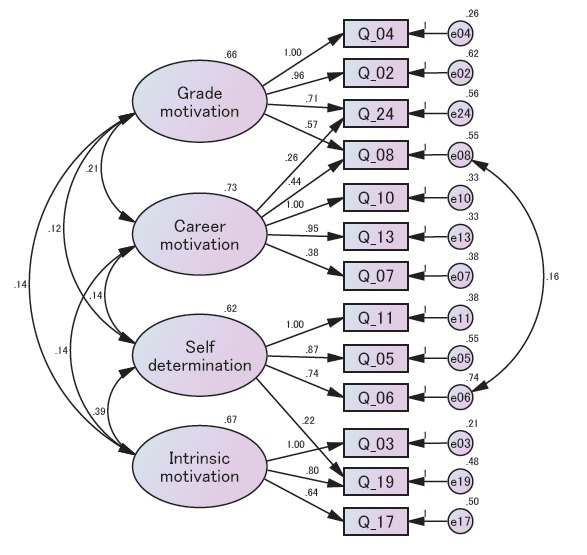
Path diagram of modified model of learning motivation among Japanese pharmacy students. Akaike information criteria=130.777; comparative fit index= 0.994; P-value of chi-square test=0.339; goodness-of-fit index=0.951; adjusted goodness-of-fit=0.919; root mean square error of approximation=0.020.

The modified model of learning motivation profile is shown in Fig. 1. All GOF statistics met the conventional criteria, as the P-values of chi-square test indicated: >0.05, CFI>0.90, GFI>0.90, AGFI>0.90, and RMSEA< 0.05.

In the modified model, variables Q_24 (“Scoring high on pharmacy tests and labs matters to me”) and Q_08 (“It is important that I get an ‘A’ in pharmacy”), originally loaded on ‘grade motivation’ in the basic model, were loaded on 2 factors of ‘grade motivation’ and ‘Career motivation.’ The pharmacy students believed that higher grades in pharmacy subjects would lead to good jobs and careers. Therefore, Q_24 and Q_08 were loaded on ‘career motivation.’ Variable Q_19 (“I enjoy learning pharmacy”) was loaded on ‘self-determined’ and ‘intrinsic motivation’ factors. Because the cognitive evaluation theory states that intrinsic motivation is one of the subscales of the self-determination theory [10], there is no conflict if some items are loaded on both ‘self-determined’ and ‘intrinsic motivation’ factors. Thus, the modified model was reasonable and showed better fit with the data than the basic model.

Using the modified model, multi-group SEM was conducted to investigate changes in the learning motivation profile over subsequent school years. The constraints model with the measurement weights and intercepts equal was the best-fitting model with CFI and RMSEA.

Table 3 shows the estimated mean values for 4 factors with test statistics and the effect size of Cohen’s d [11]. The GOFs of the model did not meet the conventional criteria [7]. The number of parameters to be estimated by multi-group analysis increases in comparison with a single-group model, suggesting that the model fit with the data in multi-group model would become worse. A CFI value larger than 0.9 usually indicates a good fit [7], but 0.85< CFI < 0.9 would be considered an acceptable or fair fit in the complex models [12].

In RMSEA, less than 0.05 would be desirable for good fit, but 0.01, 0.05, and 0.08 indicate excellent, good, and mediocre fit, respectively [13]. Based on these criteria, the multi-group model was not excellent but acceptable or fair model for the learning motivation profile of Japanese pharmacy students.

As shown in Table 3, no statistical differences were found in the estimated mean values between the 4th- and 5th-year students for all factors. The estimated mean value of ‘self-determination’ was found to increase between 4th- and 6th-year students (P= 0.048), and the effect size was medium (*d*= 0.43) [11].

## Discussion

In the established model, the component of ‘self-efficacy’ was not a factor of learning motivation among Japanese pharmacy students. Motivation based on self-efficacy is considered to rise with increase of the strength of students’ belief in their ability to accomplish the task or goal [14]. The major concern for many Japanese pharmacy students is whether they can pass the national examination for pharmacists. Since they do not have the confidence to accomplish this task, ‘self-efficacy’ is not a factor in learning motivation among pharmacy students. Thus, the model with 4 factors seems to be reasonable for learning motivation of Japanese pharmacy students.

The multi-group model was vulnerable because of an insufficient sample size but the estimated mean value for ‘self-determination’ between 4th- and 6th-year students was found to statistically increase with medium effect size [11]. These results indicate that the motivation based on ‘self-determination’ can rise after practical training programs.

Orsini et.al. [3], reported that self-determined motivation of students in health professionals education was developed by changing educational environment. They also revealed by a systematic review that a daily work of clinicians supported students’ self-determination [2]. These findings based on self-determination theory indicated that change in education environment and learning from pharmacists as practitioners at clinical sites would increase learning motivation of students. In practical training, pharmacy students are trained at clinical sites apart from the school and learn from pharmacists who work at clinical sites. The practical training would be effective in increasing self-determined motivation of Japanese pharmacy students.

Self-determination theory is known as a theory of human motivation and personality widely discussed by Deci and Ryan [10]. They claim that three psychological elements are essential in the self-determination theory: competence, relatedness, and autonomy. Since motivation based on ‘self-efficacy’ was not a factor in learning motivation of Japanese pharmacy students, the element of competence concerning ‘self-efficacy’ may not be predominant. The element of relatedness would be a more universal concept, not specific to pharmacy students. The increase of motivation based on ‘self-determination’ would result in increase of autonomy motivation. Autonomy motivation is considered to increase with the increase in flexible thinking, high-quality learning, and problem-solving skills [15]. Our findings suggest that because the students learned such skills from the pharmacists in practice during the practical training programs at clinical sites, the mean value for ‘self-determination’ increased after the practical training.

A limitation of this study is that we used a ‘science’ learning motivation questionnaire. Pharmacy students are regarded as having ‘professional-oriented’ learning motivation since they want to be healthcare professionals. Therefore, further survey using a questionnaire including a ‘professional-oriented’ factor is necessary.

## Figures and Tables

**Table 1.** Average score and standard deviation for each item and each school year among students

Component	Items	Total (n = 165)	4th-year students (n = 65)	5th-year students (n = 43)	6th-year students (n=57)	P-value by Levene’s test	P-value of pairwise comparison		
							4th—5th	4th—6th	5th—6th
1. Intrinsic motivation	Q_03. Learning pharmacy is interesting	3.53±0.94	3.48±0.89	3.60±1.16	3.53±0.83	0.099	0.771	0.955	0.911
	Q_17.1 am curious about discoveries in pharmacy	3.66±0.89	3.66±0.83	3.67±1.06	3.65±0.81	0.127	0.977	0.977	0.989
	Q_01. Learning pharmacy is relevant to my life	4.22±0.88	4.43±0.75	4.09±1.13	4.07±0.78	0.020	0.204	0.028	0.993
	Q_12. Learning pharmacy makes my life more meaningful	3.79±1.07	3.65±1.18	3.72±1.05	4.02±0.92	0.064	0.932	0.134	0.352
	Q_19.1 enjoy learning pharmacy	3.39±1.04	3.26±0.99	3.30±1.24	3.61±0.90	0.025	0.982	0.103	0.351
2. Career motivation	Q_07. Learning pharmacy will help me get a good job	4.29±0.70	4.35±0.67	4.28±0.67	4.23±0.76	0.715	0.850	0.585	0.931
	Q_13. Understanding pharmacy will benefit me in my career	3.72±1.00	3.72±1.04	3.77±1.04	3.67±0.93	0.979	0.973	0.949	0.893
	Q_10. Knowing pharmacy will give me a career advantage	3.53±1.03	3.48±1.05	3.56±1.03	3.56±1.04	0.993	0.917	0.895	1.000
	Q_25.1 will use pharmacy problem-solving skills in my career	3.78±0.85	3.77±0.81	3.70±1.01	3.84±0.77	0.043	0.920	0.867	0.717
	Q_23. My career will involve pharmacy	3.85±0.97	3.89±0.89	3.65±1.21	3.95±0.85	0.019	0.504	0.935	0.364
3. Self-determination	Q_22.1 study hard to learn pharmacy	4.03±0.80	3.98±0.80	3.98±0.89	4.12±0.73	0.775	0.999	0.610	0.640
	Q_16.1 prepare well for pharmacy tests and labs	3.70±0.95	3.65±0.93	3.67±1.11	3.77±0.87	0.198	0.988	0.749	0.869
	Q_05.1 put enough effort into learning pharmacy	3.24±1.01	3.18± 0.92	3.07±1.32	3.44±0.82	<0.001	0.873	0.245	0.247
	Q_11.1 spend a lot of time learning pharmacy	2.96±1.01	2.78±0.93	2.91±1.11	3.21±0.98	0.638	0.806	0.051	0.288
	Q_06.1 use strategies to learn pharmacy well	3.06±1.05	3.08±0.97	3.28±1.05	2.88±1.10	0.182	0.585	0.542	0.139
4. Self-efficacy	Q_18.1 believe I can earn a grade of ‘A’ in pharmacy	2.84±0.88	2.69±0.73	2.79±1.10	3.04±0.82	0.006	0.864	0.044	0.446
	Q_14.1 am confident I will do well on pharmacy labs and projects	2.60±0.96	2.35±0.82	2.33±0.89	3.09±0.99	0.641	0.986	<0.001	<0.001
	Q_15.1 believe I can master pharmacy knowledge and skills	3.60±0.94	3.46±0.89	3.42±1.10	3.89±0.82	0.012	0.975	0.016	0.050
	Q_21.1 am sure I can understand pharmacy	3.20±0.87	3.17± 0.76	3.00±1.09	3.39±0.77	0.004	0.652	0.269	0.126
	Q_09.1 am confident I will do well on pharmacy tests	2.48±0.92	2.51±0.87	2.40±1.05	2.51±0.89	0.120	0.811	1.000	0.817
5. Grade motivation	Q_04. Getting a good pharmacy grade is important to me	3.01±0.96	2.94±0.88	3.00±1.02	3.09±1.01	0.485	0.943	0.670	0.894
	Q_08. It is important that I get an “A" in pharmacy	3.28±1.00	3.25±0.97	3.37±1.09	3.25±0.97	0.453	0.799	1.000	0.807
	Q_20. I think about the grade I will get in pharmacy	3.47±1.04	3.38±0.98	3.37±1.09	3.65±1.06	0.444	0.998	0.341	0.385
	Q_24. Scoring high on pharmacy tests and labs matters to me	3.35±1.01	3.40±1.03	3.28±1.03	3.33±0.99	0.973	0.817	0.930	0.962
	Q_02.1 like to do better than other students on pharmacy tests	2.99±1.11	3.05±1.07	2.84±1.23	3.04±1.07	0.084	0.606	0.998	0.654
	1) If the assumptions of equal variances were satisfied by Levene’s test, the Tukey method was used for multiple comparisons.								
	2) If the assumptions of equal variances were not satisfied by Levene’s test, the Games-Howell method was used for multiple comparisons.								

Values are presented as mean±standard deviation.

**Table 2.** Results of factor analysis

		Factor				Degree of commonality
		1	2	3	4	
Grade motivation	Q_04	0.882	-0.090	-0.015	-0.011	0.787
	Q_02	0.738	-0.087	0.084	-0.061	0.562
	Q_24	0.591	0.163	-0.071	0.036	0.383
	Q_08	0.469	0.282	0.059	0.044	0.304
Career motivation	Q_10	-0.084	0.883	0.107	-0.069	0.804
	Q_13	0.049	0.796	-0.105	0.046	0.649
	Q_07	0.027	0.447	-0.062	0.056	0.208
Self-determination	Q_11	-0.005	0.066	0.851	-0.068	0.733
	Q_05	0.001	-0.010	0.606	0.068	0.373
	Q_06	0.061	-0.120	0.552	0.052	0.325
Intrinsic motivation	Q_03	0.054	-0.053	0.024	0.849	0.727
	Q_19	-0.093	0.029	0.176	0.649	0.462
	Q_17	-0.014	0.060	-0.076	0.640	0.419
Eigenvalues		3.800	2.312	1.448	1.073	
% of variance		29.231	17.788	11.137	8.252	
Cumulative %		29.231	47.019	58.156	66.408	
Cronbach’s alpha		0.789	0.742	0.713	0.775	

Kaiser-Meyer-Olkin measure of sampling adequacy: 0.756. Bartlett’s test of sphericity: approximate chi-square: 711.07 (df=78), P=0.000. The result of the chisquare test for goodness-of-fit statistics: 43.731 (df=32), P=0.081.

**Table 3.** Estimated mean value of learning motivation factors

Factor	4th-year students		5th-year students					6th-year students				
	m	s^2^	m	s^2^	CR	P-value	d	m	s^2^	CR	P-value	d
Grade motivation	0	0.563	-0.016	0.814	-0.085	0.932	0.02	0.081	0.612	0.514	0.607	0.11
Career motivation	0	0.782	0.047	0.847	0.240	0.810	0.05	-0.010	0.688	-0.059	0.953	0.01
Self-determination	0	0.574	0.073	0.895	0.375	0.708	0.09	0.306	0.435	1.975	0.048	0.43
Intrinsic motivation	0	0.546	0.089	1.110	0.443	0.658	0.10	0.110	0.359	0.775	0.438	0.16

Multiple-group model: all the measurement weights and intercepts in each year were constrained to be equal (comparative fit index=0.864, root mean square error of approximation= 0.055). Goodness-of-fit index and adjusted goodness-of-fit do not calculate in the multiple-group structural equation modeling. Since the estimated mean values (m) of the factors for 4th-year students were fixed at 0, the estimates expressed differences in the mean values between 4th-year students and 5th- or 6th-year students.

m, estimated mean value; s^2^, variance; CR, critical ratio; d, Cohen’s d as effect size.

## Supplementary materials

Supplement 1. Data file of the Science Motivation Questionnaire II survey to pharmacy students at Josai International University in April, 2016.

Supplement 2. Audio recording of the abstract.
